# A Rare Case of Synchronous Primary Gallbladder and Sigmoid Colon Neoplasms

**DOI:** 10.7759/cureus.34445

**Published:** 2023-01-31

**Authors:** Ilias Galanis, Ioannis Lintzeris, Magdalini Simou, Georgios Stylianidis

**Affiliations:** 1 2nd Department of Surgery, Evangelismos General Hospital, Athens, GRC

**Keywords:** biliary tract cancer, sigmoid cancer, synchronous cancers, gallbladder cancer, colonic cancer

## Abstract

Colorectal cancer is considered the third most common cancer worldwide. On the other hand, gallbladder cancer is rare. Synchronous tumors in both the colon and the gallbladder are extremely infrequent. Herein, we report the case of a female patient with sigmoid colon cancer and incidental detection of synchronous gallbladder cancer on histopathological examination of the surgical specimen. As synchronous gallbladder and colonic carcinomas are rare, physicians should be aware of these so that an optimal course of treatment can be chosen.

## Introduction

Colorectal cancer is the third most frequent type of cancer and accounts for approximately 1 out of 10 deaths caused by cancer. Gallbladder cancer is a rare type of malignancy, the fifth most common among the malignancies of the gastrointestinal tract [1]. Synchronous cancers, especially between the colon and the gallbladder, are extremely rare, with only very few cases reported in literature.

The main risk factors for the development of multiple primary cancers are genetic, immunological, environmental and iatrogenic [2]. According to Horii et al., microsatellite instabilities, due to mutations in mismatch repair genes, play a major role in the development of synchronous cancers [3]. For this reason, patients suffering from Lynch syndrome and Peutz-Jeghers syndrome are at a higher risk of synchronous billiary tract and colonic cancers [4]. Patients who have previously undergone chemotherapy or radiotherapy are also at a high risk [2]. Despite the fact that they have long been recognized as a clinical entity, the treatment and prognosis of synchronous cancers remain controversial among physicians [1]. Here, we present the case of a 75-year-old female with synchronous primary sigmoid colon and gallbladder cancer.

## Case presentation

**Figure 1:**
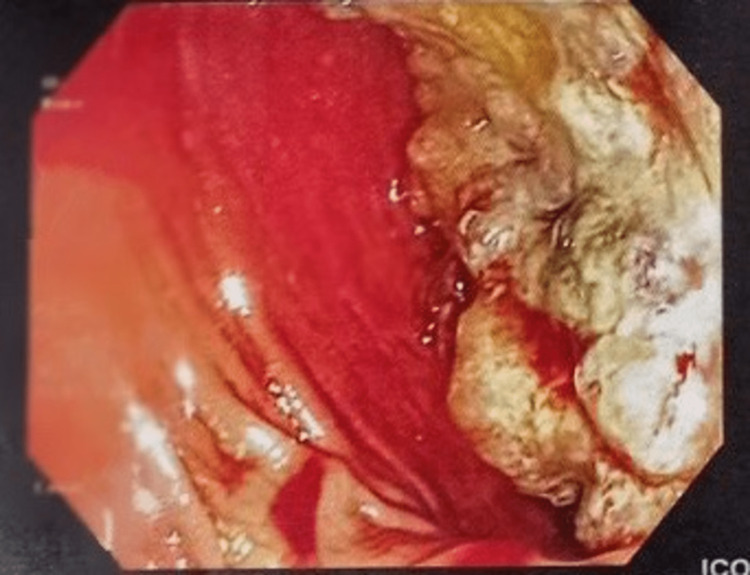
Sigmoid lesion almost obstructing the lumen

**Figure 2:**
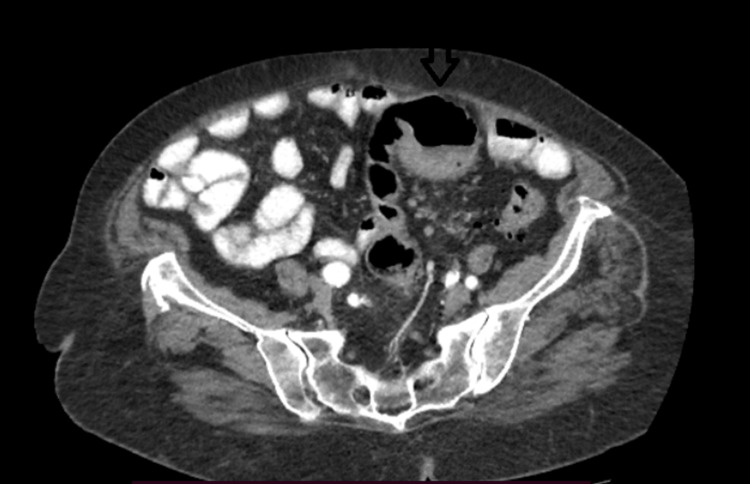
CT scan revealing distended colonic (sigmoid) loops

**Figure 3:**
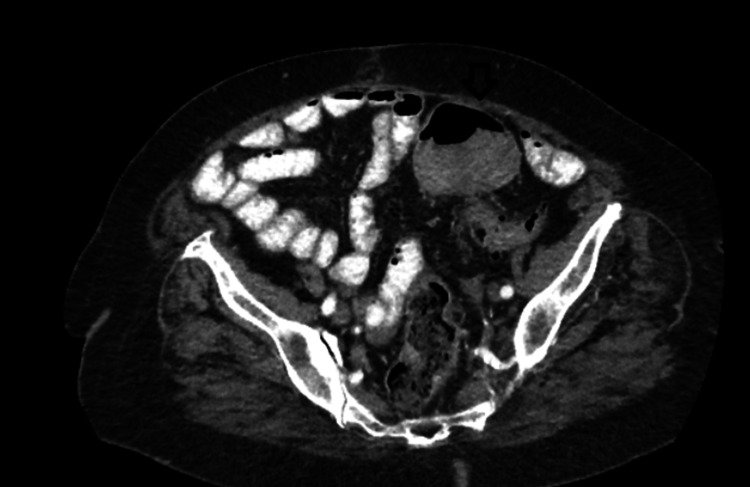
CT scan revealing distended loops and a mass almost obstructing the lumen

**Figure 4:**
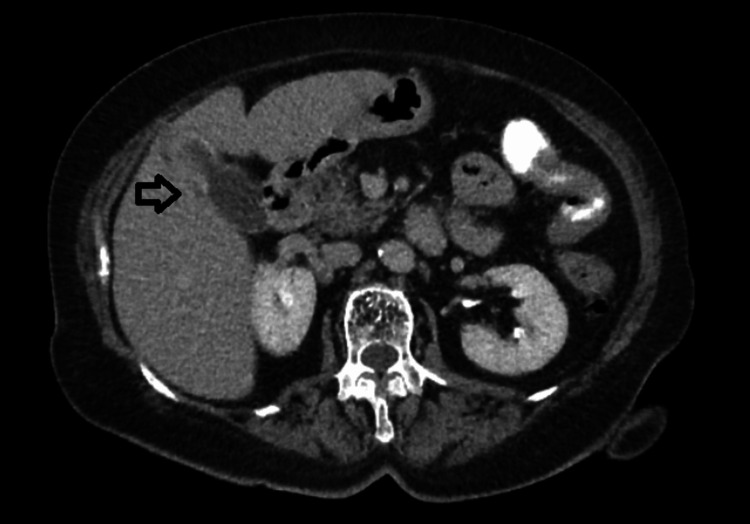
CT scan revealing a distended gallbladder with pericholecystic edema and a large gallstone

A 75-year-old female patient presented to our surgical department due to abdominal pain and hematochezia. Her past medical history included hypertension, diabetes mellitus, hypercholesterolemia and dementia. The patient was hemodynamically stable. Digital rectal examination revealed blood loss. Laboratory tests indicated low hemoglobin (10.5 mg/dl), which was the only positive laboratory finding. Colonoscopy revealed a large polypoid mass of the sigmoid colon (30 cm away from the anal verge), obstructing the lumen (Figure 1). Multiple biopsies of this lesion were taken and showed a moderately differentiated adenocarcinoma. Computed tomography of the abdomen showed the lesion of the sigmoid colon and many enlarged lymph nodes alongside the left common iliac artery (Figures 2, 3). It also revealed a gallbladder with signs of inflammation and a 2.2-cm gallstone (Figure 4).

**Figure 5:**
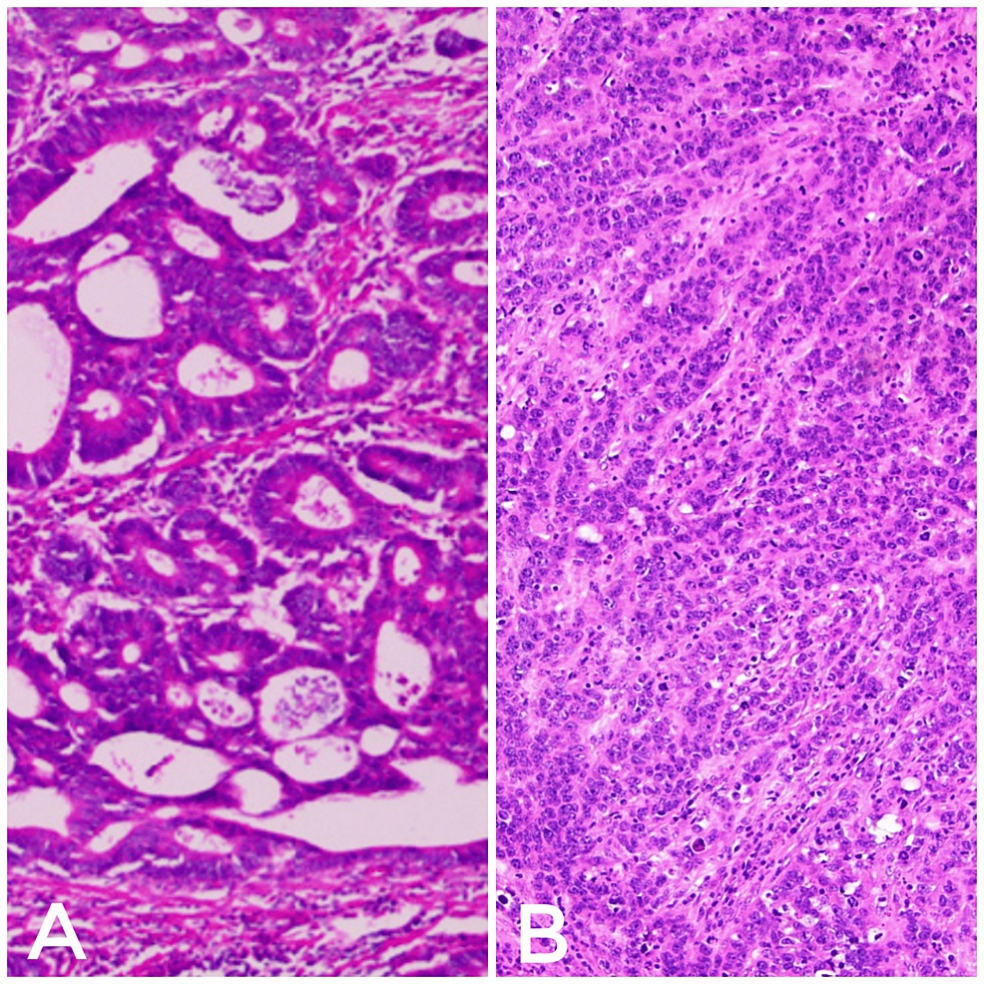
(A) Moderately differentiated adenocarcinoma of the sigmoid colon. (B) Poorly differentiated adenocarcinoma of the gallbladder

The patient underwent an open sigmoidectomy in addition to cholecystectomy. The postoperative course was uneventful and the patient was discharged on the ninth post-operative day. Colonic histopathology revealed a moderately differentiated adenocarcinoma around 6 cm in length, with free borders and without infiltration of the visceral peritoneum. Metastases were found in 13 out of 34 lymph nodes. The pathological stage for this specimen was pT3N2. Gallbladder histopathology revealed poorly differentiated adenocarcinoma, invading the adventitia with no lymph node involvement (stage pT2bN0) (Figure 5).

Due to the patient's advanced age and coexisting medical conditions, she did not undergo completion surgery. The patient was referred to the oncology department for further treatment and adjuvant therapy.

## Discussion

Billroth, in 1889, was the first to describe multiple cancers [2]. During the recent years, the occurrence of multiple primary tumors has increased, possibly because of the development of new techniques that has made their detection much easier [5]. It is crucial that the second cancer should not be mistaken as a recurrence or metastases of the other cancer. The Warren and Gates criteria, which are used to define synchronous tumors, are as follows: (1) each of the tumors must be histopathologically confirmed, (2) each must be geographically separated and distinct and the lesions should be separated by normal mucosa and (3) the probability of one being the metastasis of the other must be excluded [6]. The expression of cytokeratins CK7 and CK20 is, sometimes, used for the differential diagnosis of carcinomas originating from different sites [7].

To our knowledge, only six similar cases have been reported worldwide in the literature since 2005 [8]. However, in the last years, multiple primary tumors have been more frequently documented due to the increase in life expectancy and the improvement in diagnostic techniques. Synchronous primary gallbladder and colon cancers present with abdominal pain, weight loss and rectal bleeding along with jaundice, fever and sometimes a palpable mass of the right upper quadrant, but sometimes, these symptoms may not be present. These tumors tend to behave according to the grade and stage of each tumor separately.

There are no standard guidelines for the treatment of patients presenting with multiple malignancies. It is reported that surgical resection of each tumor, if possible, is essential [8]. The adjuvant therapy and the follow-up of these patients should follow the standard guidelines for each tumor, as their responsiveness to the chemoregimen depends on the responsiveness of each one of the neoplasms. Prognosis and survival are also defined by the stage of each tumor separately [9].

## Conclusions

Gallbladder and colonic synchronous carcinomas are a rare entity. Physicians should be aware of the possibility of synchronic tumors of the colon and the billiary tract so that they may choose the optimal treatment when this situation is highly suspected.
